# Counting the electrons in a multiphoton ionization by elastic scattering of microwaves

**DOI:** 10.1038/s41598-018-21234-y

**Published:** 2018-02-13

**Authors:** A. Sharma, M. N. Slipchenko, M. N. Shneider, X. Wang, K. A. Rahman, A. Shashurin

**Affiliations:** 1 School of Aeronautics and Astronautics, Purdue University, West Lafayette, 47906 USA; 2 School of Mechanical Engineering, Purdue University, West Lafayette, 47906 USA; 3 Department of Mechanical and Aerospace Engineering, Princeton University, Princeton, 08544 USA

## Abstract

Multiphoton ionization (MPI) is a fundamental first step in high-energy laser-matter interaction and is important for understanding the mechanism of plasma formation. With the discovery of MPI more than 50 years ago, there were numerous attempts to determine the basic physical constants of this process in direct experiments, namely photoionization rates and cross-sections of the MPI; however, no reliable data was available until now, and the spread in the literature values often reaches 2–3 orders of magnitude. This is due to the inability to conduct absolute measurements of plasma electron numbers generated by MPI, which leads to uncertainties and, sometimes, contradictions between MPI cross-section values utilized by different researchers across the field. Here, we report the first direct measurement of absolute plasma electron numbers generated at MPI of air, and subsequently we precisely determine the ionization rate and cross-section of eight-photon ionization of oxygen molecule by 800 nm photons σ_8_ = (3.3 ± 0.3)×10^−130^ W^−8^m^16^s^−1^. The method, based on the absolute measurement of the electron number created by MPI using elastic scattering of microwaves off the plasma volume in Rayleigh regime, establishes a general approach to directly measure and tabulate basic constants of the MPI process for various gases and photon energies.

## Introduction

Since the mid-1960s^1–3^ laser-induced plasmas have found numerous applications in the laboratory ranging from fundamental studies of nonequilibrium plasmas^4,5^, soft ionization in mass spectroscopy^6^, development of compact particle accelerators^7,8^, and X-ray and deep UV radiation sources^9,10^ to diagnostic techniques such as laser-induced breakdown spectroscopy and laser electronic excitation tagging^11–13^. In addition, the laser-induced plasma is related to studies of various nonlinear effects at beam propagation, such as laser pulse filamentation, laser beam collapse, self-trapping, dispersion, modulation instability, pulse splitting, etc.^5,11,12,14,15^.

Multiphoton ionization (MPI) is a key first step in all laser-induced plasma. However, basic physical constants of the MPI process, namely photoionization rates and cross-sections, have never been precisely determined in direct experiments. This refers to the fact that there are no diagnostic tools available today to provide absolute measurements of the total number of electrons in plasma volume *N*_*e*_ or local plasma density distribution *n*_*e*_(**r**) generated by femtosecond laser pulse in a relatively low intensity linear regime. For MPI of atmospheric air, plasma density has to be below *n*_*e*_ ≤ 10^15^–10^16^ cm^−3^ to ensure that the contribution of plasma nonlinearities to the refraction index is negligible^5^. At the same time, the sensitivity of laser interferometry is limited to *n*_*e*_ ≥ 10^16^–10^17^ cm^−3^ due to the minimal measurable shifts of the interference fringes^16–18^. A number of semi-empirical methods for relative measurements of plasma density were proposed, but all require absolute calibration based upon theoretically predicted values of plasma number density. Time-of-flight (TOF) mass spectrometer measurements of ion currents generated by laser-induced plasma filament have been conducted to measure photoionization rates^14,19–21^. The fundamental limitation of this technique is the inability to conduct absolute calibration of the system since the reference object against which the calibration can be completed is not readily available. Therefore, the TOF mass spectrometer measurements relied on theoretical estimation of total number of electrons in the focal zone in order to conduct absolute calibration of the system. Very recently, scattering of THz radiation from laser-induced plasmas was proposed for spatially unresolved relative measurements of *n*_*e*_^16,17^. Other recently proposed measurement techniques were based on measurements of capacitive response times of the system, including a capacitor coupled with laser-induced plasma loaded inside^22–24^. These attempts to measure *n*_*e*_ in laser-induced plasmas were characterized by varying degrees of success and reliability of the obtained data, but none of them provided an ultimate solution for absolute plasma density measurements.

Analysis of various theoretical and semi-empirical approaches undertaken previously led to a large variability of photoionization process constants available in the literature, some of which were controversial. For example, photoionization rates for O_2_ reported by Mishima in ref.^25^ are approximately 2 orders of magnitude higher than that reported by Talebpour in ref.^20^. In addition, comparison of photoionization rates for N_2_ and O_2_ yields to 3 orders of magnitude higher photoionization rates for O_2_ compared to that of N_2_ due to difference in ionization potentials (15.576 eV and 12.063 eV respectively)^24^, while experimentally determined photoionization rates reported by Talebpour for N_2_ and O_2_ yields doubtful proximity: 1.5 × 10^9^ s^−1^ for N_2_ and 3 × 10^9^ s^−1^ for O_2_ for the laser intensity 3 × 10^13^ W/cm^2^ ^20^.

Therefore, photoionization rates and cross-sections of the MPI process still remain unknown 50 years after the discovery. Large discrepancies in photoionization rates available in the literature cause contradictory conclusions and are generally disadvantageous for theoretical modeling of a wide class of problems involving laser-induced plasmas. In this work, we propose a direct experimental approach to measure the total number of electrons created at the MPI of air and directly determine the ionization rate and cross-section of MPI for the oxygen molecule. The proposed approach has tremendous fundamental significance and great potential for applications, since it paves the way to directly measure and tabulate basic constants of the MPI process for various gases and photon energies.

## Methodology of MPI cross-section determination

Ionization of gas in laser-induced plasma is associated with multiphoton (MPI) and tunneling processes, two limiting cases of essentially the same physical process of nonlinear photoionization. The choice of the governing mechanism is dictated by Keldysh parameter *γ*, defined as a ratio of laser frequency *ω* to tunneling frequency *ω*_*t*_ characterizing time of electron tunneling through the potential barrier: $\gamma =\frac{\omega }{{\omega }_{t}}=\frac{\omega \sqrt{2m{ {\mathcal E} }_{i}}}{eE}$, where *E*- amplitude of incident electric field,$\,{ {\mathcal E} }_{i}$- ionization potential, and *m* and *e* are electron mass and charge, respectively. In the case of low frequency limit (and/or large laser intensity) *ω* < *ω*_*t*_, the electron has sufficient time to tunnel through the barrier and ionization is driven by the tunnel effect, while for high-frequency limit (and/or low laser intensity) *ω* > *ω*_*t*_, the electric field varies faster than the time required for tunneling and ionization is governed by the MPI process.

In our method, the cross-section of the MPI is determined experimentally based on absolute measurement of total electron numbers (*N*_*e*_) generated by a femtosecond laser pulse and precise measurements of the laser pulse characteristics. The experiments have been conducted at low laser intensities (<2.7 ×10^13^ W/cm^2^, as detailed below) in order to ensure a pure linear operation regime when nonlinearities associated with plasma creation and optical Kerr effect are negligible (see below for details). In this case, plasma formation due to MPI by the femtosecond laser is described by the simple differential equation $\frac{\partial {n}_{e}}{\partial t}=\nu \cdot ({n}_{0}-{n}_{e})$, where *n*_*e*_ - plasma density, *ν* = *σ*_*m*_*I*^*m*^ – ionization rate, *σ*_*m*_ – cross-section of *m*-photon ionization process with $m=\,{\rm{Int}}(\frac{{ {\mathcal E} }_{i}}{\hslash \omega })+1$, *I*– local instantaneous value of laser field intensity, and *n*_0_ – background gas density, while other physical processes can be neglected on the extremely fast time scale of the laser pulse^1,26^. This equation can be easily integrated and plasma density *n*_*e*_ created as result of action of femtosecond laser pulse can be found: ${n}_{e}={n}_{0}(1-{e}^{-\int vdt})$. For the case of low ionization degree *n*_*e*_ ≪ *n*_0_, plasma density distribution immediately after the laser pulse can be written in the form:

1 $${n}_{e}({\bf{r}})={n}_{0}\int \nu dt={\sigma }_{m}{n}_{0}\int I{({\bf{r}},t)}^{m}dt$$

where time integration is taken over the duration of the laser pulse at particular location **r**. Total electron number *N*_*e*_ generated by the laser pulse can be expressed by integrating Equation (1) over the entire plasma volume:

2 $${N}_{e}={\sigma }_{m}{n}_{0}\iint I{({\bf{r}},t)}^{m}dtdV$$

Equation (2) provides a general expression that can be used to determine the MPI cross-section as follows. Total electron numbers (*N*_*e*_) generated by the femtosecond laser pulse are measured using the Rayleigh Microwave Scattering (RMS) technique (see Methods for details). Spatial and temporal intensity distribution *I*(**r**, *t*) are determined in precise measurements of the laser beam and the integral in the right-hand side is calculated. Then, one can determine *σ*_*m*_ from Equation (2) for the known background gas density *n*_0_.

General expression (2) can be simplified if additional assumptions are made. Firstly, we will consider in this work the most practical case of Gaussian beam. In this case, spatial and temporal intensity dependences can be expressed as follows:

3 $$I(r,z,t)={I}_{0}\frac{{w}_{0}^{2}}{w{(z)}^{2}}{e}^{-\frac{2{r}^{2}}{w{(z)}^{2}}}{e}^{-\frac{{(t-{t}^{\ast })}^{2}}{{\tau }^{2}}}$$

where *I*_0_ – intensity in the beam center, ${w}_{0}-1/{e}^{2}$ waist radius (at *z* = 0), $w(z)={w}_{0}\sqrt{1+{(\frac{z}{{z}_{R}})}^{2}}-1/{e}^{2}$ beam radius at location *z* along the beam, *z*_*R*_ - Rayleigh length and *τ* – characteristic temporal width of the beam. This approximation uses the standard for the non-dispersing medium Gaussian beam optics spatial distribution $({I}_{{\bf{r}}}(r,z)={I}_{0}\frac{{w}_{0}^{2}}{w{(z)}^{2}}{e}^{-\frac{2{r}^{2}}{w{(z)}^{2}}})$ with Gaussian temporal shape ${e}^{-\frac{{(t-{t}^{\ast }(r,z))}^{2}}{{\tau }^{2}}}$, where t^*^ = t^*^(*r*, *z*) indicates moment of time when the beam reaches particular (*r*, *z*)-location (it is taken as a given that beam peak reaches the waist at *z* = 0 at time *t* = 0, so that t^*^(*r*, 0) = 0 and t^*^(0, *z*) = *z*/*c*)^27,28^. All parameters of the beam in Equation (3), namely *I*_0_, *w*_0_, *z*_*R*_, and *τ*, are determined experimentally.

Secondly, we will consider in this work the case of atmospheric air and 800 nm laser. In this case, MPI of oxygen molecules is the dominant process since the O_2_ photoionization rate is 2–3 orders larger than that for N_2_ due to its lower ionization potential^5,24^. Thus, by using ionization energy of oxygen molecule ${ {\mathcal E} }_{i}$ = 12.2 eV and energy of 800 nm ionizing photons of *ħω* = 1.55 eV, it is clear that the eight-photon photoionization process should be considered, namely *m* = 8.

A simplified form of the expression (2) for MPI of air with femtosecond laser pulse of Gaussian shape in temporal and spatial domains can be deduced by analytical integration of the intensity in form (3), namely: ${\int }^{}{\int }^{}I{(r,z,t)}^{8}dVdt=\frac{231\pi }{1024\cdot 16}\sqrt{\frac{\pi }{8}}{{I}_{0}}^{8}\pi {w}_{0}^{2}{z}_{R}\tau$ (see Supplementary Materials), and plugging it into the right-hand side of Equation (2). Finally, Equation (2) can be reduced to the form:

4 $${N}_{e}=\frac{231\pi }{1024\cdot 16}\sqrt{\frac{\pi }{8}}{\sigma }_{8}{n}_{0}\tau \pi {w}_{0}^{2}{z}_{R}\cdot {{I}_{0}}^{8}$$

The determination of MPI cross-section of oxygen is conducted using Equation (4) as follows. *N*_*e*_ in the left-hand side of the equation is measured using the Rayleigh Microwave Scattering (RMS) technique (see Methods). Spatial and temporal characteristics of the laser beam (*I*_0_, *w*_0_, *z*_*R*_, and *τ*) in the right-hand side of Equation (4) are measured directly. Then, the experimental dependence of *N*_*e*_ vs. laser intensities *I*_0_ is plotted, and *σ*_8_ is determined by obtaining the best fit of that dependence using Equation (4).

## Experimental Details

**Figure 1:**
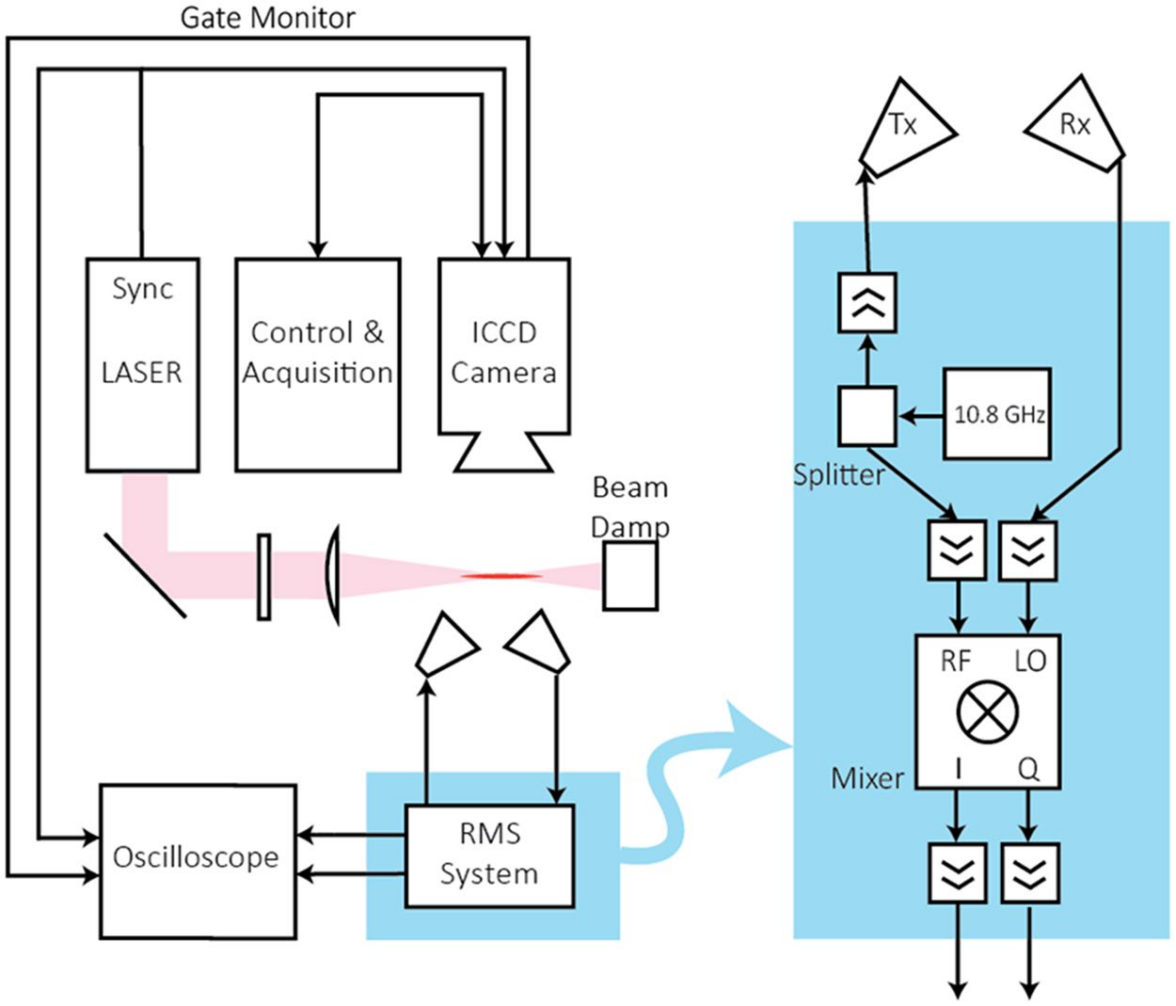
Experimental setup and RMS homodyne system. A 800 nm Ti:Sapphire femtosecond laser operating at a repetition rate of 100 Hz was focused using a 1000 mm plano-convex lens to create the plasma. A homodyne-type Rayleigh Microwave Scattering system calibrated against dielectric scatterers was used to measure the absolute number of electrons in the plasma volume.

The experimental layout including femtosecond laser and RMS system are shown schematically in Fig. 1. Photoionization of air (relative humidity 30%, temperature about 300 K) was achieved by focusing laser pulses having Gaussian temporal and spatial shape from a 800 nm Ti:Sapphire laser of 164 fs FWHM having repetition rate of 100 Hz using a 1000 mm plano-convex lens. The laser repetition rate was decreased from nominal *f*_*rep*_ = 1 kHz to ensure no “memory effect” in the interrogated volume. To this end, the number of electrons generated by the laser pulse was measured as a function of the laser repetition rate (using Rayleigh Microwave Scattering as detailed below). The number of electrons being generated was independent on the repetition rates for *f*_*rep*_ ≤ 100 Hz indicating absence of “memory effects,” while an increase was observed at higher repetition rates. The diameter of the incident beam on the lens was 7 mm. The pulse energy was varied using a linear polarizer and measured using a laser power meter (Gentec-EO XLP12-3S-H2-DO). Images of the plasma were taken using a 1024i Pi Max 4 ICCD camera. Coordinate *z* = 0 was chosen at the beam focal plane of the strongly attenuated laser beam (no plasma present). Several experiments were also conducted using 400 mm lens. The results similar to that with 1000 mm lens we obtained; however, focal length increase to 1000 mm led to increase of the total number of electrons generated by the laser due to increase of Rayleigh length and beam waist. This increased the accuracy of the measurements with Rayleigh Microwave Scattering system, and therefore, 1000 mm lens was used in the experiments presented below.

Measurements of the total number of electrons in the plasma volume were conducted using Rayleigh Microwave Scattering (RMS) diagnostics in which absolute calibration was completed using dielectric scatterers with known physical properties (see Methods). A homodyne-type RMS system operating at the microwave frequency 10.8 GHz was used, as shown schematically in Fig. 1. The microwave signal from the source was split in two arms. One arm sent microwaves to the plasma using the radiating horn, while the second arm delivered the signal directly to the LO-input of the *I/Q* mixer. Microwave radiation was linearly polarized along the plasma channel orientation. Radiating and detecting horns were mounted at a distance of 6 cm from the plasma. The signal scattered from the plasma was received by the detecting horn, amplified, and sent to the RF-input of the *I/Q* mixer. The two outputs of the *I/Q* mixer were again amplified and captured on the oscilloscope. All components of the microwave system operated in the linear range of powers to ensure a measured response proportional to the amplitude of signal scattered from the plasma volume. The overall time response of the system was measured to be about 250 ps.

### Measurements of MPI cross-section of oxygen

**Figure 2:**
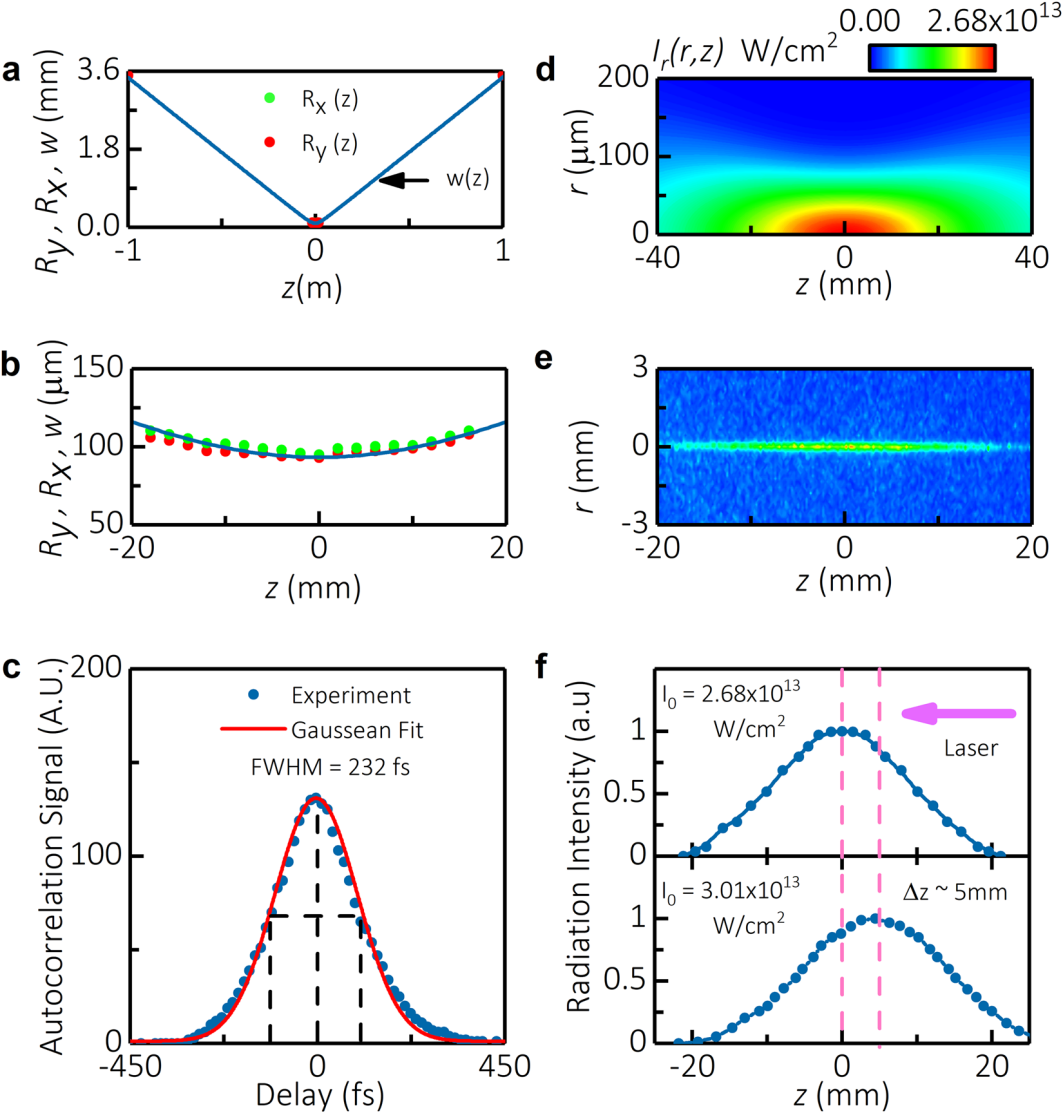
Measurements of the laser beam properties for intensity in the center *I*_0_ = 2.68 × 10^13^ W/cm^2^. (**a**) $1/{e}^{2}$ radius of the laser beam at various *z*-locations measured using beam profiler (green and red points) and the beam waist approximated by analytical function $w(z)={w}_{o}\sqrt{1+{(z/{z}_{R})}^{2}}$ (solid blue curve). (**b**) Measured and approximated beam radius in the vicinity of the beam waist. (**c**) Temporal shape of the beam determined from intensity autocorrelation function measurements and approximated by Gaussian fit. (**d**) 2D spatial distribution of the laser beam intensity approximated by Gaussian fit ${I}_{r}(r,z)={I}_{0}\frac{{w}_{0}^{2}}{w{(z)}^{2}}{e}^{-\frac{2{r}^{2}}{w{(z)}^{2}}}$ based on measured laser beam radius and Rayleigh length. (**e**) Photographs of laser-induced plasma taken at an exposure time of 10 ns by ICCD camera. (**f**) Distribution of intensity radiated by the plasma plotted along the z-axis for two laser intensities.

The spatial distribution of laser beam intensity was determined using beam profiler measurements conducted with a strongly attenuated laser beam. To this end, a set of attenuator plates was used to reduce the beam intensity manifold (about 2–3 orders of magnitude) to completely eliminate plasma creation. The $1/{e}^{2}$ radius of the beam, measured using the beam profiler at various *z*-locations, is shown in Fig. 2(a,b). Location of beam waist refers to coordinate *z* = 0. Waist radius in *x* - and *y* -directions were *R*_*x*_ = 92.17 μm and *R*_*y*_ = 94.41 μm, respectively. The average radius of the beam (*w*) was chosen to satisfy condition *πw*^2^ = *πR*_*x*_*R*_*y*_ at each measured *z*-location and was approximated by analytical function $w(z)={w}_{0}\sqrt{1+{(\frac{z}{{z}_{R}})}^{2}}$ with waist radius *w*_0_ = 93.28 μm and Rayleigh length *z*_*R*_ = 26.98 mm to achieve the best fit of the experimental data. Based on the data fit the beam quality factor M^2^ = 1.3, which is slightly worse than expected M^2^ = 1.1 from the laser and is attributed to the laser alignment and use of spherical lenses for beam size control.

The temporal shape of the laser pulse was determined using measurements of intensity autocorrelation function by means of a second harmonic generation crystal. The autocorrelation function had a nearly Gaussian shape with full width at half maximum (FWHM) equal to *FWHM*_*τ*_ = 232fs, as shown in Fig. 2(c). Thus, it may be concluded that laser intensity in the time domain was also Gaussian, with $FWH{M}_{t}=\frac{232\,{\rm{fs}}}{\sqrt{2}}=164\,{\rm{fs}}$. Finally, temporal dependence of the laser intensity was approximated by Gaussian distribution $I\propto {e}^{-\frac{{t}^{2}}{{\tau }^{2}}}$ with $\tau =\frac{FWH{M}_{t}}{2\sqrt{\mathrm{ln}\,2}}=98.6\,{\rm{fs}}.$

Measured temporal and spatial parameters of the femtosecond laser pulse utilized in this work are summarized in Table 1. Mean values averaged over the multiple measurements of the corresponding quantities and their standard errors are shown in the Table 1.

**Table 1:** Measured time-space parameters of the laser pulse.

*w*_0_	1/e^2^ waist radius	93.3 ± 0.7 μm
*z*_*R*_	Rayleigh length	26.9 ± 0.3 mm
*τ*	Temporal width	98.6 ± 5.2 fs

Optical images of the laser-induced plasma created by MPI of air were analyzed to demonstrate that nonlinear effects in the non-attenuated laser beam were small and, thus, intensity approximation used in Equation (3) still applies when the plasma was on. A typical photograph of the laser-induced plasma taken by ICCD camera (exposure time *t* = 0–10 ns) is shown in Fig. 2(e) (energy in pulse 620 μJ, intensity at the beam center *I*_0_ = 2.68 × 10^13^W/cm^2^). Figure 2(f) shows the corresponding distribution of plasma radiation intensity (*S*) along the *z*-axis for two laser intensities *I*_0_ = 2.68 × 10^13^W/cm^2^ and 3.01 × 10^13^W/cm^2^. It was observed that the focal plane of the beam coincided with coordinate *z* = 0 for *I*_0_ ≤ 2.68 × 10^13^W/cm^2^. A shift of the focal plane toward the direction of the laser was observed for higher intensities, which can be explained by action of focusing Kerr nonlinearity. Based on that experimental evidence, we have concluded that nonlinear effects (Kerr and plasma nonlinearities) were negligible for *I*_0_ ≤ 2.68 × 10^13^W/cm^2^.

**Figure 3:**
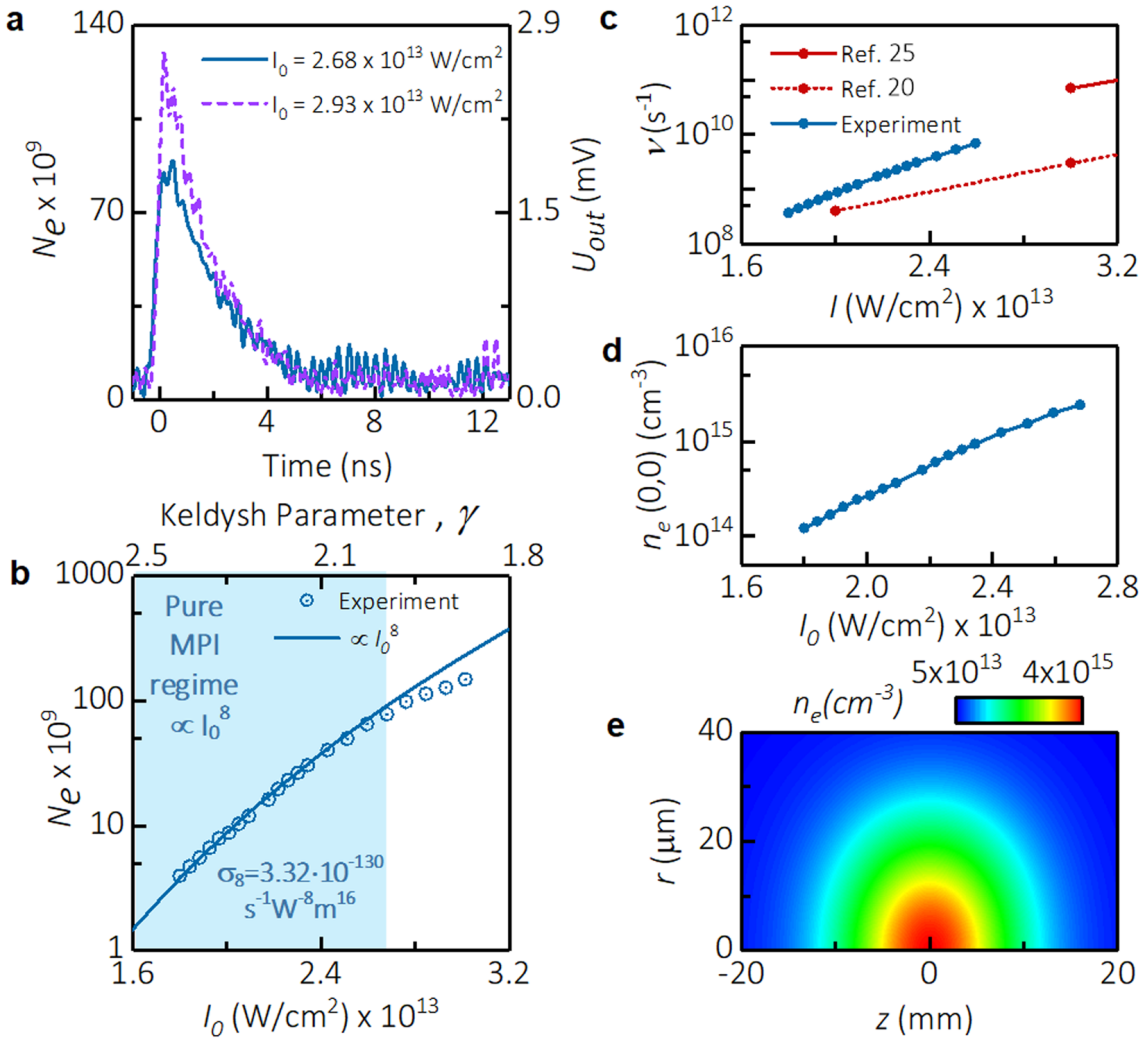
Absolute measurements of parameters of MPI of air. (**a**) Temporal evolution of microwave signal scattered from the plasma *U*_*out*_(*t*) and total number of electrons in plasma volume *N*_*e*_(*t*). (**b**) Measured dependence of *N*_*e*_ immediately after the plasma creation vs. intensity at the beam center *I*_0_. (**c**) Comparison of theoretical and semi-empirical literature data with directly measured photoionization rate in this paper. The blue shaded area identifies range of intensities where pure MPI was observed *N*_*e*_ ∝ ${{I}_{0}}^{8}$. (**d**) Plasma density at the beam center *n*_*e*_(0, 0) immediately after the laser pulse vs. intensity *I*_0_. (**e**) 2D distribution of plasma density immediately after the laser pulse for *I*_0_ = 2.68 × 10^13^W/cm^2^.

The electron number generated by fs-laser laser pulse was measured using the RMS system shown in Fig. 1. Figure 3(a) presents a typical temporal evolution of the number of electrons and amplitude of scattered microwave signal for two values of intensity: *I*_0_ = 2.68 × 10^13^ W/cm^2^ and 2.93 × 10^13^W/cm^2^. Right and left vertical axes indicate the signal directly measured by the RMS and the total number of electrons in the plasma volume *N*_*e*_ determined using the approach described in Methods. It was observed that plasma decayed faster for larger laser intensities; specifically, two-fold decay occurs on characteristic times 2.5 ns and 2 ns for *I*_0_ = 2.68 × 10^13^ W/cm^2^ and 2.93 × 10^13^W/cm^2^, respectively. Several experiments were also performed in conditions of higher humidity (relative humidity 65%). Higher humidity had no effect on the yield of electrons, but resulted in faster plasma decay.

Two distinct physical processes occurring on significantly different time scales can be traced on Fig. 3(a). The first process is the fast rise at the moment of plasma creation (around *t* = 0) associated with the laser pulse passing the waist region and reaching the peak value of *N*_*e*_(*t*). The characteristic time of plasma creation is about the time required for light to pass the Raleigh length around the beam waist, namely $\frac{{z}_{R}}{c} \sim 0.1\,{\rm{ns}}$. The second process is the decay of the plasma remaining after the laser pulse, which occurs on characteristics times of about several nanoseconds, according to Fig. 3(a) (see section below). The RMS diagnostic was unable to temporally resolve the precise details of plasma creation due comparable response times of the system used (about 0.25 ns). However, the RMS system precisely measured *N*_*e*_peak value and the following plasma decay *N*_*e*_(*t*) since plasma recombination is occurring on a time scale several nanoseconds slower and, therefore, the difference between the true peak value and the measured value is negligible. Note that the maximal electron number occurring immediately after the plasma creation is denoted as *N*_*e*_ throughout the manuscript, while decay of the plasma refers to the dependence *N*_*e*_(*t*). Figure 3(b) presents the experimentally measured dependence of *N*_*e*_ immediately after the plasma creation versus intensity at the beam center *I*_0_. This peak value *N*_*e*_is used for the purpose of determination of MPI cross-section *σ*_8_.

We will now determine MPI cross-section *σ*_8_ by fitting the measured dependence of *N*_*e*_ versus *I*_0_ shown in Fig. 3(b) using analytical expression (4). According to the analytical expression, *N*_*e*_ increases with laser intensity as ${{I}_{0}}^{8}$. RMS data shown in Fig. 3(b) indicates that dependence ${N}_{e}\propto {{I}_{0}}^{8}$ was satisfied with high accuracy at low intensities *I*_0_ < 2.7×10^13^ W/cm^2^, which represents a clear manifestation of the pure MPI regime. Deviation from the ${{I}_{0}}^{8}$-law for higher intensities indicates a departure from the pure MPI process at these higher *I*_0_, which can be explained by relative proximity of Keldysh parameter *γ* to 1 [top horizontal axis of the Fig. 3(b)] and action of Kerr nonlinearity. Therefore, *N*_*e*_ was fitted by the ${{I}_{0}}^{8}$-law for intensities *I*_0_ < 2.7 × 10^13^ W/cm^2^, and MPI cross-section *σ*_8_ was determined based on the fit of this initial segment of the dependence as shown by the blue line in Fig. 3(b) using the parameters of the laser system measured above and density of molecular oxygen in the background air *n*_0_ ≈ 5.13 × 10^18^ cm^−3^. Finally, the MPI cross-section was determined to be *σ*_8_ = (3.3 ± 0.3) × 10^−130^ W^−8^m^16^s^−1^.

### Photoionization rates, electron and species’ densities

We will now consider oxygen photoionization rates based on the measured data and compare it with data available in the literature. For laser beam center intensities *I*_0_ < 2.7 × 10^13^ W/cm^2^ (pure MPI regime), dependence of photoionization rate can be readily plotted as *ν* = *σ*_8_*I*^8^, shown by the solid blue curve in Fig. 3(c), using the value of *σ*_8_ obtained above. The comparison with previously available data determined based on theoretical and semi-empirical approaches is also shown in Fig. 3(c)^20,24^. It is clear that semi-empirical predictions given in ref.^20^ underestimated the photoionization rates about 2–3 times, while purely theoretical predictions in ref.^24^ seem to slightly overestimate the rates.

The experiments conducted here pave the way to determining plasma density distribution created in the fs-laser-induced plasmas. Distribution of the plasma density immediately after the laser pulse for the laser Gaussian intensity distribution used in this work can be written using Equation (1) as: $${n}_{e}(r,z)={\sigma }_{8}{n}_{0}$$$${\int }^{}I{(r,z,t)}^{8}dt={n}_{e}(0,0)\frac{{w}_{0}^{2}}{w{(z)}^{2}}{e}^{-\frac{2{r}^{2}}{w{(z)}^{2}}}$$. Integrating the left and right side of this expression relates the plasma density at the origin location immediately after the laser pulse *n*_*e*_ (0, 0) with the directly measured quantities of *N*_*e*_, *w*_0_and *z*_*R*_ as follows: $${n}_{e}(0,0)=\frac{{N}_{e}}{\frac{231\pi }{1024\cdot 16}\pi {w}_{0}^{2}{z}_{R}}$$. Figure 3(d) shows the dependence of *n*_*e*_(0, 0) on laser intensity. A 2D distribution of plasma density *n*_*e*_(*r*, *z*) for *I*_0_ = 2.68 × 10^13^ W/cm^2^ is shown in Fig. 3(e).

We have numerically simulated the plasma decay to validate our experimental measurements. Plasma decay was simulated using a 1D axially symmetric model in radial direction that self-consistently integrates Navier-Stokes, electron heat conduction, and electron-vibration energy transfer equations^25^. The model accounts for recombination of molecular ions, attachment processes, formation and decay of complex ions, electron energy losses due to electronic, vibrational excitations, and elastic scattering.

**Figure 4:**
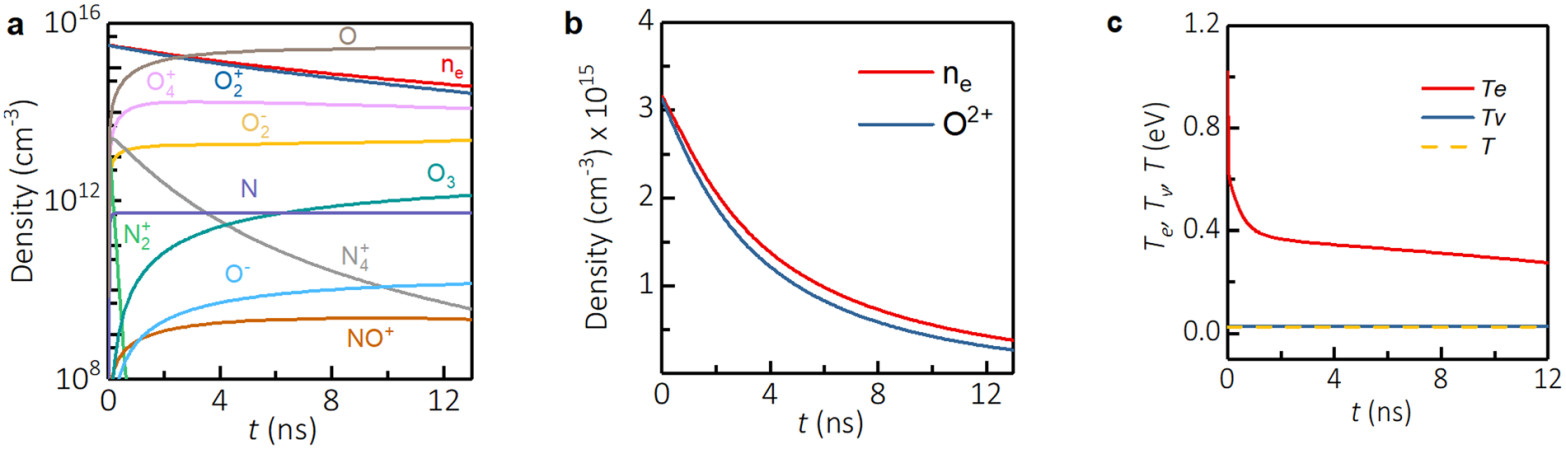
Numerical simulations of plasma decay. Plasma decay after fs-laser pulse for *I*_0_ = 2.710^13^W/cm^2^. (**a**) Plasma species, (**b**) expanded view of electrons and O^2+^, and (**c**) electron, vibrational, and gas temperatures.

The simulated decay of the densities and temperatures of various plasma species at the point of origin is shown in Fig. 4 for intensity in the beam center *I*_0_ = 2.7 × 10^13^W/cm^2^. An extremely fast (<1 ns) decrease of electron temperature to about 0.4 eV is associated with electron energy loss due to vibrational excitation of molecules, while slower later decay is governed by elastic collisions. Plasma density in the center decayed twice in about 3.2 ns, primarily dominated by dissociative and three-body recombination of molecular ions. Slightly faster plasma decay times observed in the experiments (about 2.5 ns) might be related to the presence of water vapor in the ambient air.

## Concluding remarks

In this work, we have presented a methodology that is paving the way for precise determination of the physical constants of multi-photon ionization, namely cross-section and ionization rate. The method utilizes precise measurement of the spatial and temporal distributions of the laser beam intensity and absolute measurement of total electron number in the plasma volume by means of elastic scattering of microwaves off the plasma volume and absolute calibration of the microwave system using dielectric scatterers. We have demonstrated the capability of this method on the example of eight-photon ionization of molecular oxygen and determined the corresponding MPI cross-section to be *σ*_8_ = (3.3 ± 0.3) × 10^−130^ W^−8^m^16^s^−1^. Future studies may focus on the precise tabulation of the cross-sections and photoionization rates of the multi-photon ionization for different gases and laser wavelengths using the methodology proposed and validated in this work. This effort would provide critical experimental evidence for the theoretical modeling of laser-induced plasmas.

## Methods

### Rayleigh Microwave Scattering method description

**Figure 5:**
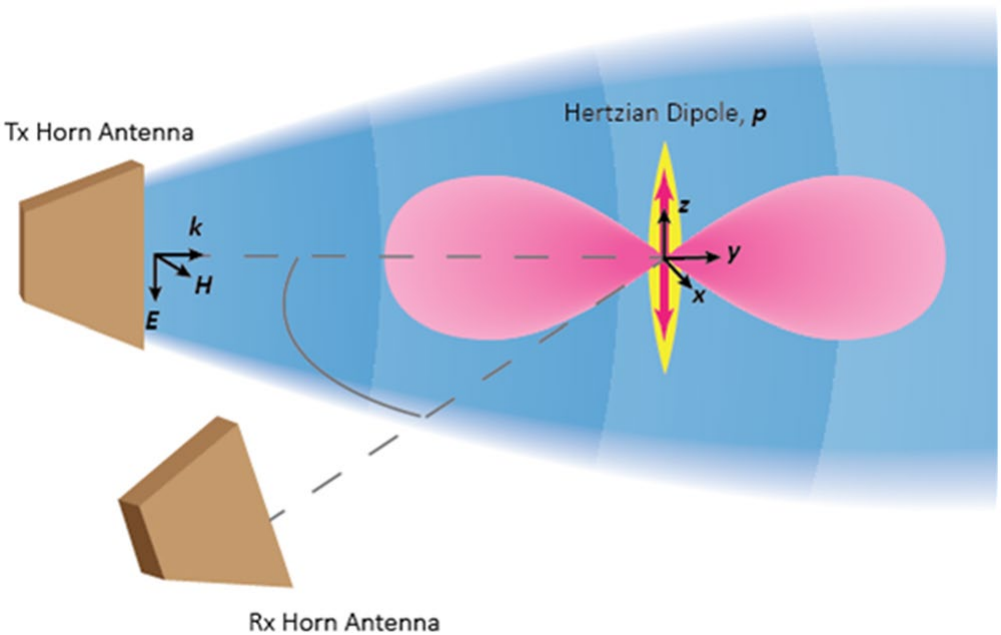
Detailed schematics of the Rayleigh Microwave Scattering (RMS) system.

To more clearly illustrate the method of absolute measurement of electron number in plasma volume, we present the schematics of the Rayleigh Microwave Scattering (RMS) system in Fig. 5 and provide a more detailed description here. In the Rayleigh Microwave Scattering technique, elastic scattering of microwave radiation off the plasma volume is measured and the total number of electrons in the plasma volume is determined. The scattered radiation is created as the result of polarization of the plasma channel in the external microwave field. For the thin plasma channel, when amplitude of the microwave field is nearly uniformly distributed inside the plasma, the radiation in far-field is equivalent to the Hertz dipole radiation. Overall, such a process is analogous to elastic Rayleigh scattering of light, when radiation wavelength significantly exceeds the scatterer size. The signal scattered from the plasma is proportional to the total electron numbers in the plasma volume. Absolute calibration of the RMS system was conducted using dielectric scatterers with known physical properties.

Linearly polarized microwave radiation at frequency of 10.8 GHz was scattered on the collinearly oriented plasma filament and the amplitude of the scattered signal was measured. Microwaves are radiated and detected using a horn (as shown in Fig. 5) mounted at a distance 6 cm from the plasma. A homodyne-type detection system was used for the scattered microwave signal measurements by means of an I/Q mixer.

We first demonstrate that the amplitude of the electric field induced inside the scatterer channel as a result of irradiation with microwaves was uniform throughout the channel and equal to the field amplitude *E*_0_ in the incident wave. Slender prolate plasma channel geometry with length (*l*) significantly exceeding the diameter (*d*) was used in this work. For conditions of this experiment, the plasma channel can be considered thin compared to skin depth (so that *f*[*GHz*] $\le \frac{2.5}{\sigma [{\Omega }^{-1}c{m}^{-1}]\cdot d{[mm]}^{2}}$)^29^. In this case, the amplitude of the electric field induced inside the scatterer with dielectric permittivity *ε* and conductivity *σ* can be written as $E=\frac{{E}_{i}}{\sqrt{{(1+k(\varepsilon -1))}^{2}+{(k\frac{\sigma }{{\varepsilon }_{0}\omega })}^{2}}}$, where *k* - depolarization factor governed by the channel geometry, and *E*_*i*_ – amplitude of incident microwave electric field at the channel location^30–32^. For the conditions of the experiments conducted here, the depolarization factor *k* is small due to large aspect ratio *AR* = *l/d* ≫ 1: *k* ≈ (1)/(*AR*^2^)ln(*AR*) ≪ 1^29–31^. Therefore, the amplitude of the electric field inside the channel is close to that in the incident wave *E* = *E*_*i*_^31,33,34^. The flatness of the wave front surface along the scatterer length was ensured by placing the plasma scatterer at distance $r > \frac{{l}^{2}}{\lambda }\approx \,1\,{\rm{c}}{\rm{m}}$.

Electrons in the plasma volume experience oscillations with an amplitude of $s=\frac{e{E}_{i}}{m\,\omega }$ due to the incident microwave field (we consider no restoring force due to prolate plasma channel geometries)^35^. The electron collision frequency in the denominator is governed by those with gas particles (*ν*_*eg*_) for densities <10^17^ cm^−3^, since contribution of electron-ion collisions can be neglected in that range, so that finally $s=\frac{e{E}_{i}}{{m}_{eg}\omega }$. Electron-gas collision frequency *ν*_*eg*_ is independent on plasma density, ensuring that electrons in each location inside the plasma volume experience essentially the same displacement. We used *ν*_*eg*_ = 5.18 × 10^11^ s^−1^ based on electron-gas elastic collision cross-section *σ*_*eg*_ = 5 × 10^−16^ cm^−2^ and electron temperature *T*_*e*_ = 0.4 eV.

Total dipole moment of the plasma channel (*p*) can be calculated as follows:

5 $$p=es\int n(r,z)2\pi r\,dr\,dz=es{N}_{e}=\frac{{e}^{2}}{m\nu }\frac{{E}_{i}}{\omega }{N}_{e}$$

Radiation from the plasma dipole in a plane perpendicular to the dipole orientation was detected by the same horn antenna as incident one (see Fig. 5). The antenna was placed at a distance *r* = 6 cm from the plasma channel to ensure the dominant contribution of far-field $( \sim \frac{{k}^{2}p}{r})$, while near-field $( \sim \frac{p}{{r}^{3}})$ is negligible (*kr* > 6)^32,36^. Thus, the amplitude of the electric field at the location of the detecting horn was:

6 $${E}_{s}=\frac{{k}^{2}p}{r}=\frac{{e}^{2}}{m{c}^{2}\nu }\frac{\omega {E}_{i}}{r}{N}_{e}$$

A homodyne-type detection system was used for the scattered microwave signal measurements, which provides output voltage ${U}_{out}\propto {E}_{s}$. The detection was achieved by means of an *I/Q* Mixer, providing in-phase (*I*) and quadrature (*Q*) outputs. The total amplitude of the scattered microwave signal is determined as:$\,{U}_{out}=\sqrt{{I}^{2}+{Q}^{2}}$. The amplifiers and the mixer used in the microwave system operate in a linear mode for the entire range of the scattered signal amplitudes, thereby ensuring that the output signal *U*_*out*_ is proportional to the electric field amplitude of scattered radiation *E*_*s*_ at the detection horn location: ${U}_{out}\propto {E}_{s}$.

7 $${U}_{out}\propto {E}_{s}=\frac{{e}^{2}}{m{c}^{2}\nu }\frac{\omega {E}_{i}}{r}{N}_{e}$$

It is clear that the RMS system detects the total number of electrons in the plasma volume accurate to the coefficient function of the specific microwave system used. Absolute calibration of the RMS can be conducted using dielectric scatterers with known physical properties. To this end, we now consider RMS system signal generated by the prolate scatterer made of dielectric material with dielectric constant *ε* and volume *V*. The only difference from the above consideration for the plasma channel would be that total dipole moment induced in the scatterer is *p* = *ε*_0_(*ε* − 1)*E*_*i*_*V*, and thus:

8 $${U}_{out}\propto {E}_{s}=\frac{{\varepsilon }_{0}(\varepsilon -1)}{{c}^{2}}\frac{{\omega }^{2}{E}_{i}}{r}V$$

One particularly convenient form of expression for the measured output of the RMS system is:

9 $${U}_{out}=\{\begin{array}{l}A\cdot \frac{{e}^{2}}{m}{N}_{e}-{\rm{for}}\,{\rm{plasma}}\,\\ A\cdot V\cdot {\varepsilon }_{0}(\varepsilon -1)\omega -{\rm{for}}\,{\rm{dielectric}}\,{\rm{bullet}}\end{array}$$

where *A* –proportionality coefficient, which is a property of the specific microwave system (utilized components, geometry, microwave power, etc.) while independent of scatterer properties, and it can be found using scatterers with known properties. The lower part of Equation 9 was used for calibration of the specific microwave system, particularly in order to determine the value of coefficient *A*. Cylindrical dielectric bullets made of Teflon with diameter 3.175 mm and length 1 cm were shot through the microwave field (along the same axis where plasma was later placed) using a pneumatic gun with velocities below 100 m/s in order to generate a time-varying response on the dielectric bullet passage for separation of signal scattered from the bullet from one DC background caused by reflections from surroundings, elements of microwave circuit, etc.

**Figure 6:**
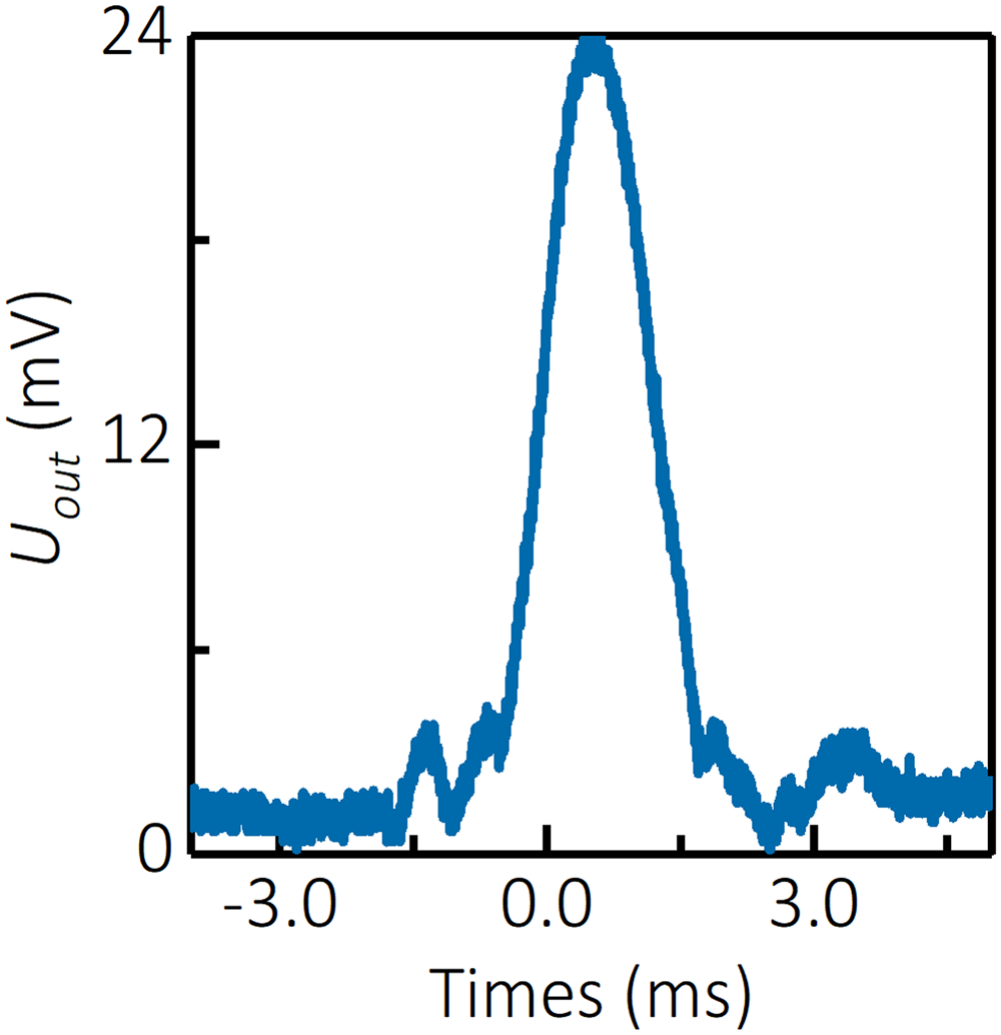
Calibration of the Rayleigh Microwave Scattering (RMS) system.

Calibration of the RMS system with a 1 cm long and 3.175 mm diameter Teflon bullet yielded the response of the RMS system shown in Fig. 6. Coefficient A was found to be A = 2.12 × 10^5^ V·Ω·m^−2^ in this calibration procedure. Thus, the relation between the total electron number in the plasma volume *N*_*e*_ and amplitude of the scattered system measured using the RMS system *U*_*out*_ was: *N*_*e*_ = *U*_*out*_ × 4.81 × 10^13^.

### Data availability

The data that support the plots within this paper and other findings of this study are available from the corresponding author upon reasonable request.

## Electronic supplementary material

Supplementary Info
