# Supplementary material for: Counting the electrons in a multiphoton ionization by elastic scattering of microwaves
Source: Sci Rep. 2018 Feb 13;8:2874. doi: 10.1038/s41598-018-21234-y (PMC5811500; doi:10.1038/s41598-018-21234-y)
Supplement: Supplementary file 1 — Supplementary Info [file 41598_2018_21234_MOESM1_ESM.pdf]

Counting the electrons in a multiphoton ionization by elastic scattering of microwaves

A. Sharma, M.N. Slipchenko, M.N. Shneider, X. Wang, K.A. Rahman, A. Shashurin

Supplementary Materials

Calculating of intensity integral

We present calculation of laser intensity integral over spatial and temporal variables:

$$\int\int I(r,z,t)^8 dtdV = \frac{231\pi}{1024\cdot 16}\sqrt{\frac{\pi}{8}}{I_0}^8 \pi w_0^2 z_R \tau$$

The integration is first taken over time period when laser pulse exists at particular location of space and then spatial integral is taken over region where laser beam presents. For the specific system utilized in current experiment, main contribution to the temporal integral is gained during the laser pulse time of τ=98.6 fs, while spatial integral is accumulated primarily in vicinity of the beam waist where intensity is maximal.

The limits of the integrals can be extended to infinity when analytical approximation space-time dependence of the laser pulse in the form $I(r,z,t) = I_0 \frac{w_0^2}{w(z)^2} e^{-\frac{2r^2}{w(z)^2}} e^{-\frac{(t-t^*(r,z))^2}{\tau^2}}$ with $w(z) = w_0\sqrt{1+\left(\frac{z}{z_R}\right)^2}$ is used:

$$\int\int I(r,z,t)^8 dtdV = \int_{-\infty}^{+\infty}\int_{0}^{+\infty}\int_{-\infty}^{+\infty} {I_0}^8 \frac{w_0^{16}}{w(z)^{16}} e^{-\frac{16r^2}{w(z)^2}} e^{-\frac{8(t-t^*(r,z))^2}{\tau^2}} 2\pi r\, dt\, dr\, dz$$

Inner temporal integral can be calculated analytically by changing of variables to $\tilde{t} = \frac{\sqrt{8}(t-t^*(r,z))}{\tau}$ for any finite (r,z)-location $\int_{-\infty}^{+\infty} e^{-\frac{8(t-t^*(r,z))}{\tau^2}} dt = \frac{\tau}{\sqrt{8}}\int_{-\infty}^{+\infty} e^{-\tilde{t}^2} d\tilde{t} = \sqrt{\frac{\pi}{8}}\tau$. The outer spatial integral can be first simply calculated over r – variable and then using standard integral $\int_{-\infty}^{+\infty} \frac{1}{[1+x^2]^7} dx = \frac{231\pi}{1024}$. This finally yields:

$$\int\int I(r,z,t)^8 dtdV = {I_0}^8\sqrt{\frac{\pi}{8}}\tau \int_{-\infty}^{+\infty}\int_0^{+\infty} \frac{w_0^{16}}{w(z)^{16}} e^{-\frac{16r^2}{w(z)^2}} 2\pi r dr dz = {I_0}^8\sqrt{\frac{\pi}{8}}\tau \int_0^{+\infty} \frac{\pi w_0^{16}}{16}\frac{1}{w(z)^{14}} dz =$$
$${I_0}^8\sqrt{\frac{\pi}{8}}\tau \frac{\pi w_0^2}{16}\int_{-\infty}^{+\infty} \frac{1}{\left[1+\left(\frac{z}{z_R}\right)^2\right]^7} dz = {I_0}^8\sqrt{\frac{\pi}{8}}\tau \frac{\pi w_0^2}{16}\frac{231\pi}{1024} z_R$$
